# Supplementary material for: Priming of lineage-specifying genes by Bcl11b is required for lineage choice in post-selection thymocytes
Source: Nat Commun. 2017 Sep 26;8:702. doi: 10.1038/s41467-017-00768-1 (PMC5615048; doi:10.1038/s41467-017-00768-1)
Supplement: Supplementary file 1 — Supplementary information [file 41467_2017_768_MOESM1_ESM.pdf]

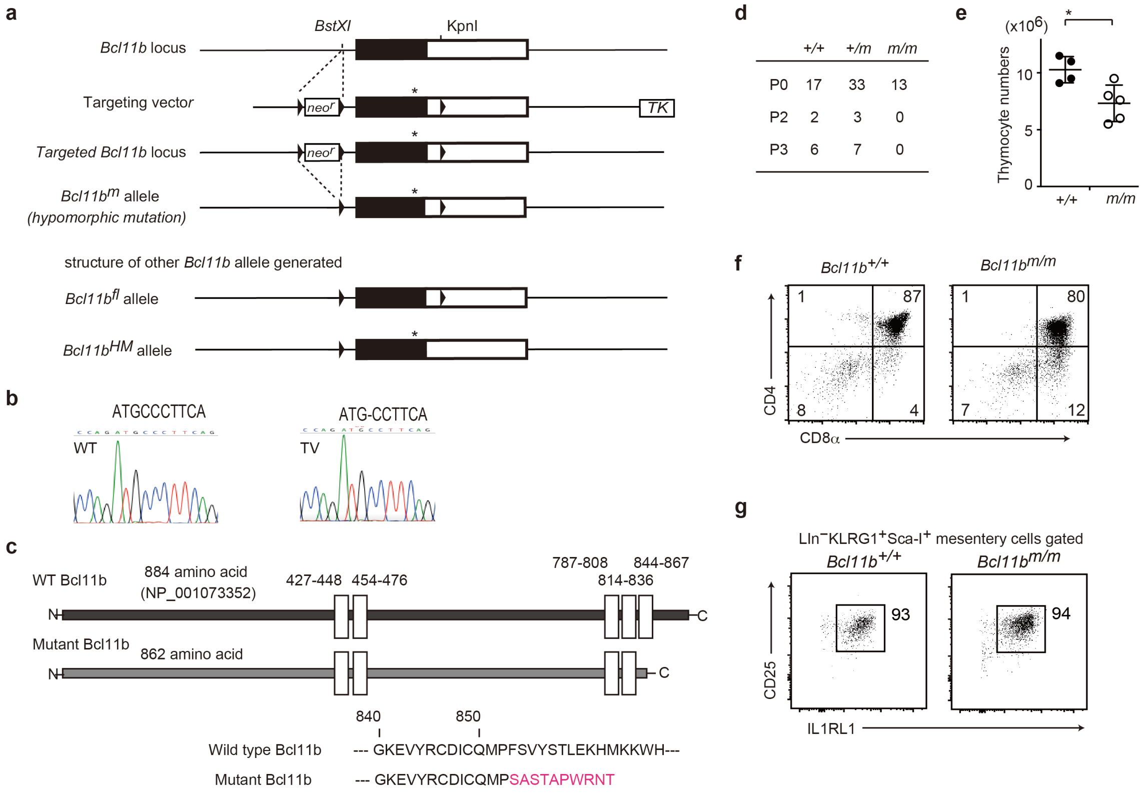

Supplementary Figure 1. Generation of a *hypomorphic Bcl11b* mutant allele. (a) Schematic of the strategy to generate mutant *Bcl11b* alleles by gene targeting and structures of mutant *Bcl11b* alleles generated. Filled and open boxes represent coding and 3' untranslated region (3'UTR) in the exon 4 of the murine *Bcl11b* gene. Triangle and asterisk represent loxP sequences and the single nucleotide deletion, respectively. (b) Sequence of the targeting vector (TV) and the genome fragment (WT) obtained from a phage library indicated that the single nucleotide deletion, which was marked as * in (a), was incorporated into the targeting vector during sequential ligation steps. (c) Predicted structure of mutant Bcl11b protein from the $Bcl11b^{m}$ allele. The C2H2 zinc finger motifs are shown as open boxes with their corresponding amino acid numbers. Aberrant amino acid sequences in mutant $Bcl11^{HM}$ protein are shown in red font (bottom). (d) Genotypes of offspring obtained from the $Bcl11b^{+/m}$ intercrossing at day 0 (P0), day 2 (P2) and day3 (P3) after birth. (e) Absolute numbers of total thymocytes in control (●) and $Bcl11b^{m/m}$ (○) neonates. Means ± S.D. * $P < 0.05$ (unpaired *t* test). (f) Dot plots showing CD4/CD8 expression in total thymocytes from newborn $Bcl11b^{+/+}$ and $Bcl11b^{m/m}$ mice. In contrast to Bcl11b-deficient mice, thymocyte development was not arrested at the DN2 stage in the $Bcl11b^{m/m}$ mice. Data are representative of at least five mice. (g) Development of type-2 innate lymphoid cells (ILC2) from $Bcl11b^{+/+}$ and $Bcl11b^{m/m}$ progenitors in $Il2rg^{-/-}$:$Rag1^{-/-}$ host mice. ILC2s were defined as $CD25^{+}IL1RL1^{+}$ cells in the $Lin^{-}KLRG1^{+}Sca\text{-}1^{+}$ population from the mesentery. Representative result of two host mice.

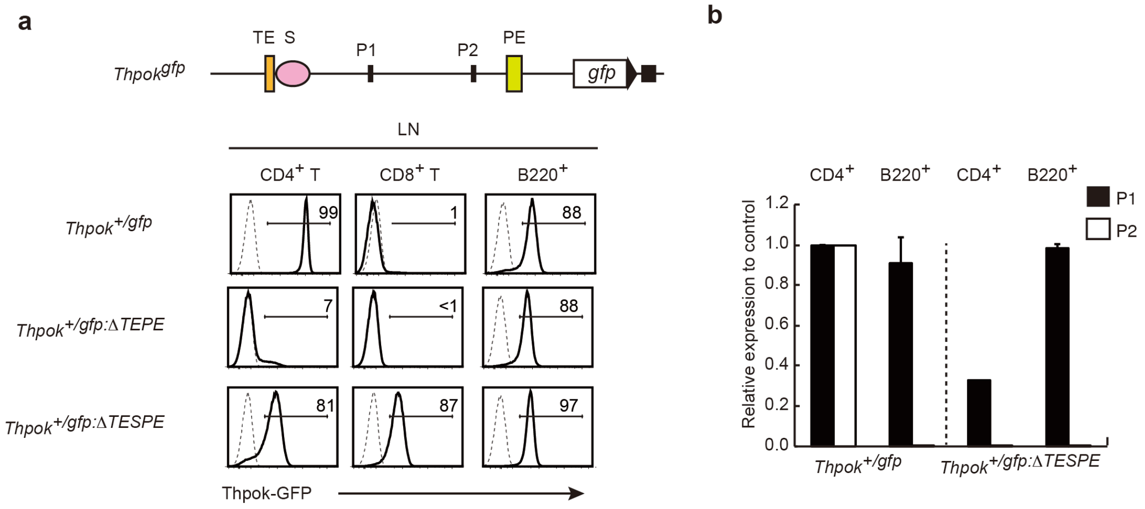

Supplementary Figure 2. Expression of Thpok-gfp from the *$Thpok^{gfp:\Delta TEPE}$* and *$Thpok^{gfp:\Delta TESPE}$* allele in lymph node cells. (a) Schematic structure of *$Thpok^{gfp}$* allele and positions of three regulatory elements, *thymic enhancer* (TE), *Thpok silencer* (S) and *proximal enhancer* (PE). Histograms showing Thpok-GFP expression in indicated lymph node (LN) cells from the *$Thpok^{gfp}$*, *$Thpok^{gfp:\Delta TEPE}$* and *$Thpok^{gfp:\Delta TESPE}$* allele. Dotted line is Thpok-GFP expression from *$Thpok^{+/+}$* cells. Numbers in dot plots indicate percentage of Thpok-GFP expressing cells. One representative of at least three independent mice. (b) Quantitative RT-PCR for distal P1 (■) and proximal P2 (□) promoter-derived *Thpok-gfp* mRNA amount in $CD4^{+}$ T and $B220^{+}$ B cells from *$Thpok^{gfp}$* and *$Thpok^{gfp:\Delta TESPE}$* allele. Levels of P1- and P2-promoter-specific transcripts relative to those in $CD4^{+}$ T cells from the *$Thpok^{gfp}$* allele is shown. One representative of two independent experiments.

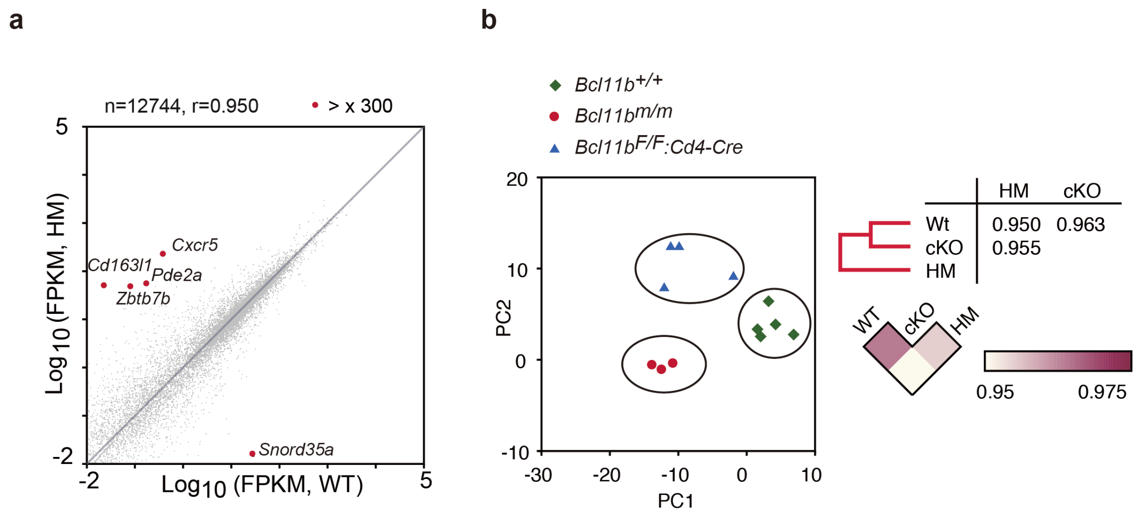

Supplementary Figure 3. Gene expression profiling by RNA-seq. (a) RNA-seq analyses comparing gene expression profiles derived from at least three independent samples of CD24^{hi}TCRβ^{lo} pre-selection *Bcl11b*$^{+/+}$ (WT) and *Bcl11b*$^{m/m}$ (HM) thymocytes. (b) Principle component analyses showing relationship of WT, *Bcl11b*$^{m/m}$ (HM) and *Bcl11b*$^{fl/fl}$*:Cd4-Cre* (cKO) pre-selection DP thymocytes.

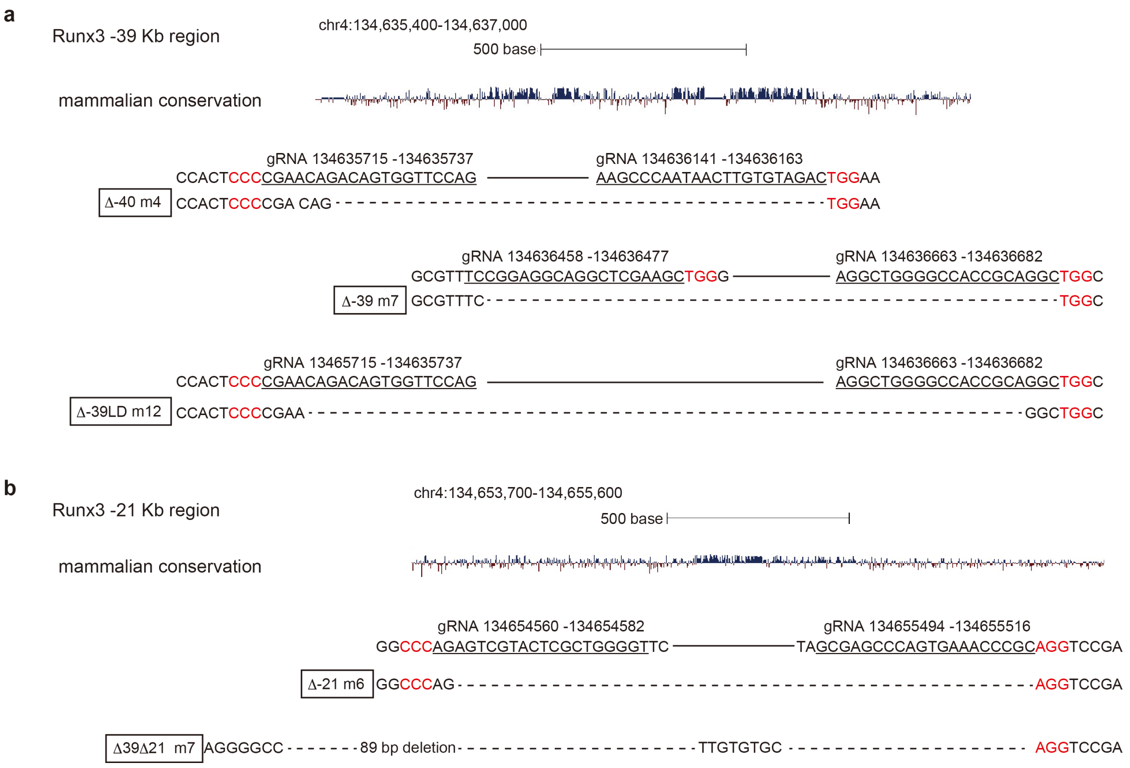

Supplementary Figure 4. Removal of putative enhancers from the *Runx3-tdTomato* locus by CRISPR/Cas9-mediated genome editing. (a and b) Top bar showing homology around -39 kb (a) and -21 kb (b) regions upstream of the distal *P1-Runx3* promoter in mammals. Regions deleted in mutant mouse strains are indicated as dotted lines. Underlined and red font indicate protospacer and PAM sequence, respectively. In the Δ39Δ21 m7 line, TTGTGTGC sequences were inserted.

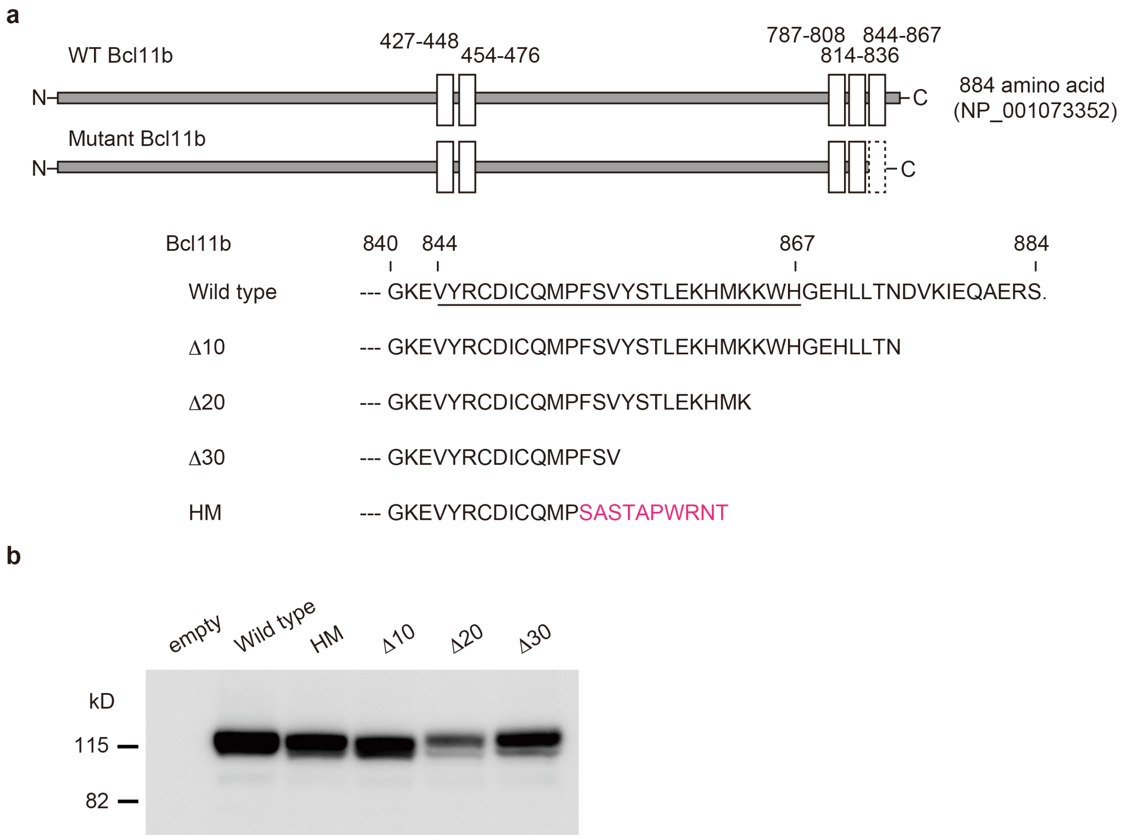

Supplementary Figure 5. Expression of mutant Bcl11b proteins from retroviral vectors. (a) Schematic structures of wild-type and mutant Bcl11b proteins lacking C-terminal end sequences. C2H2 zinc finger motifs are shown as open boxes. The C-terminal end amino acid sequences of each Bcl11b mutant proteins are indicated with that of hypomorphic (HM) Bcl11b mutant protein. Sequences corresponding to the last zing-finger domain (844 - 867 a.a.) are underlined. Aberrant amino acid sequences in mutant Bcl11b^{HM} protein are shown in red font. (b) Western blot using an antibody that reacts with anti-N-terminal Bcl11b sequences showing expression of each mutant Bcl11b protein in the packaging cell line, Plat-E.

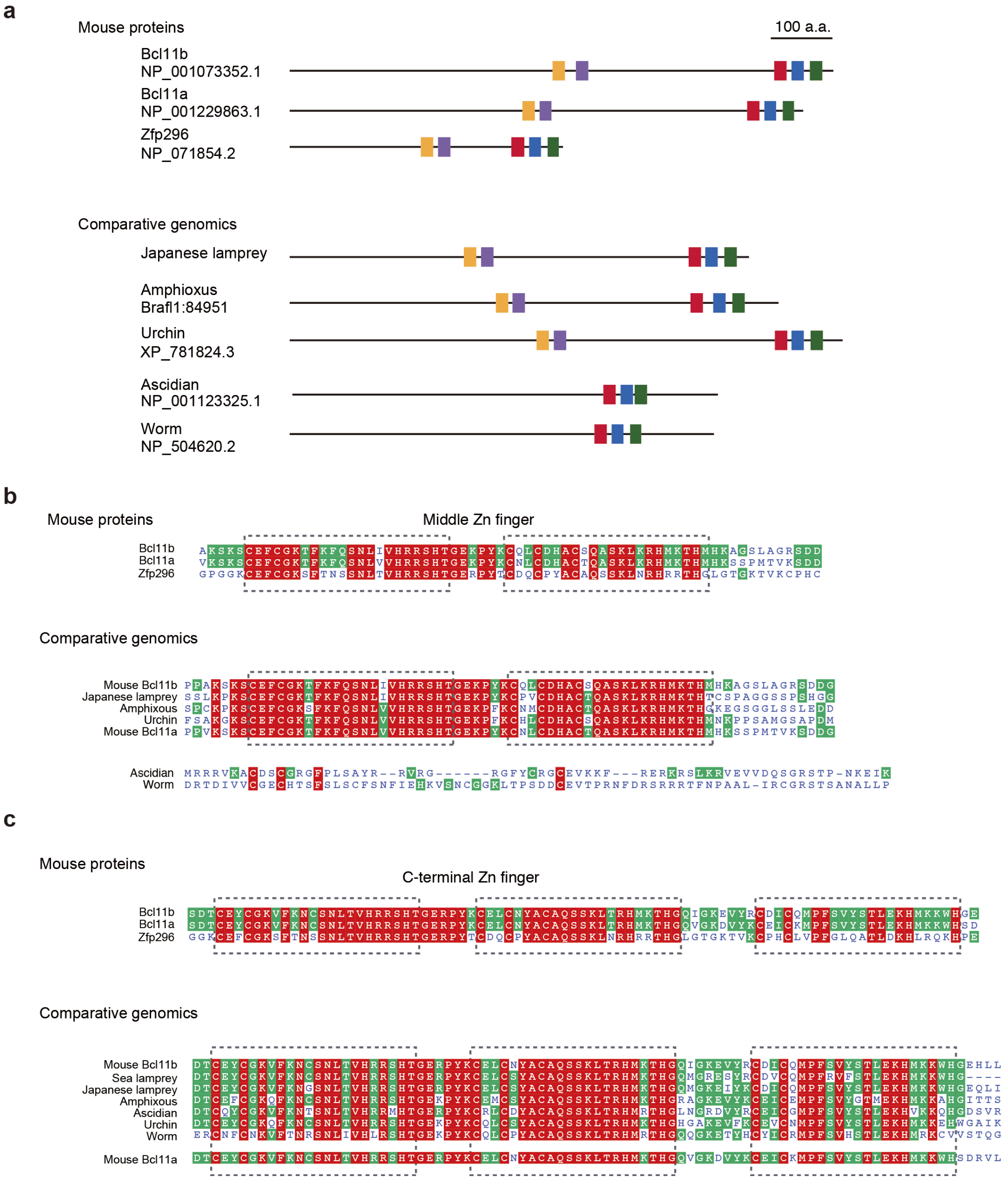

Supplementary Figure 6. Evolutionary conservation of zinc-finger motifs in the Bcl11 family. (a) Schematic structures of Bcl11related proteins and Bcl11 orthologues in several species. In mouse, only Zfp296 possesses the zinc-finger motifs similar to Bcl11a and Bcl11b. Ascidian (*C. intestinalis*) and worm (*C. elegans*), have a Bcl11-related protein that lacks the middle two zinc-finger motifs. (b and c) Alignment of amino acid sequences around the middle two zinc-finger motifs (b) and the C-terminal triplet zinc-finger motifs (c) of Bcl11 orthologues from several species. Each zinc-finger is indicated as a dashed box.

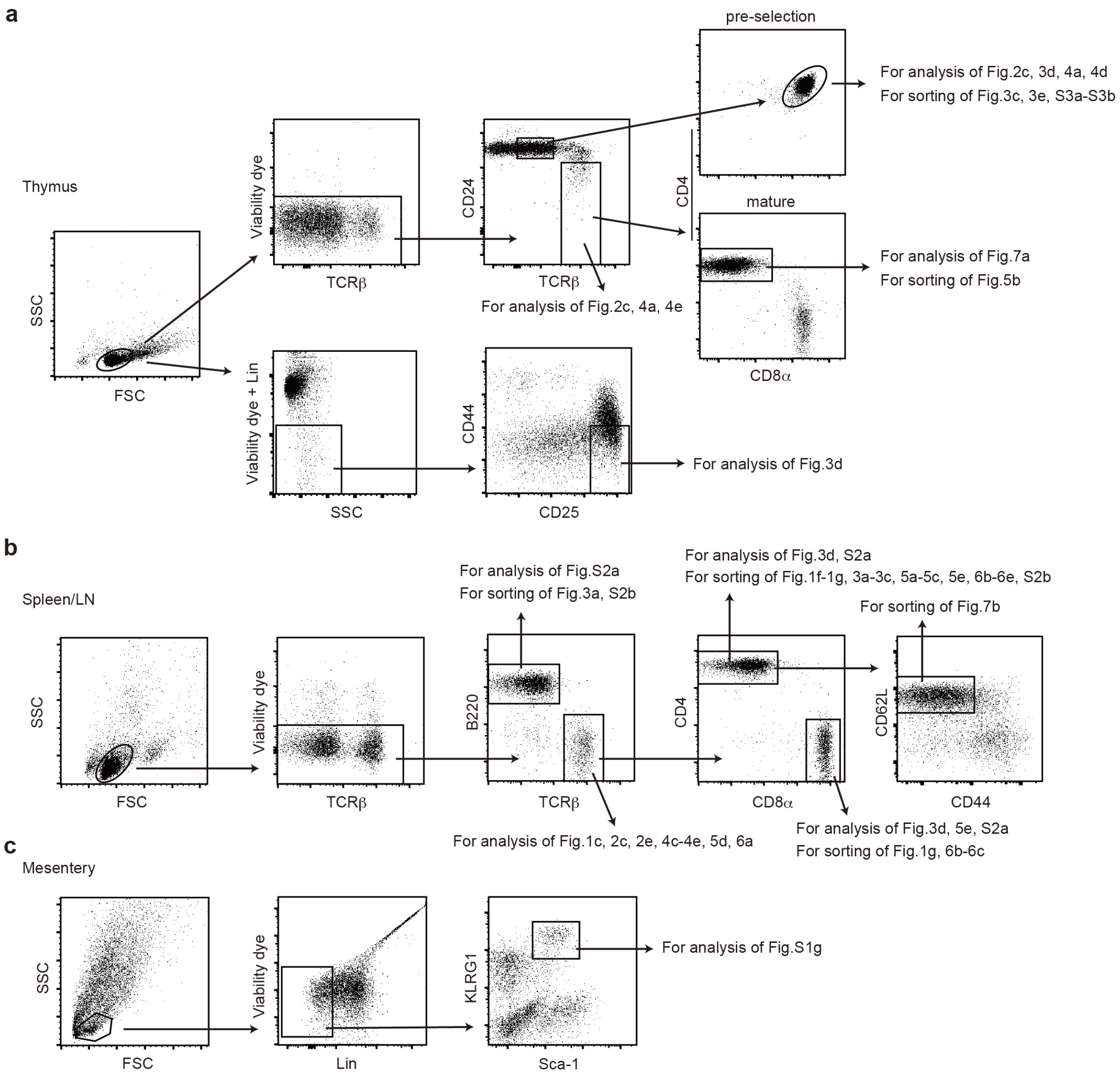

Supplementary Figure 7. Gating strategies for cell analyses and sorting. Representative FACS plots showing: Gating strategy for analysis and sorting of the thymocytes (a) and peripheral lymphoid population (spleen and lymph node) (b). (c) Gating scheme for analysis of mesenteric ILC2.

Figure 1a

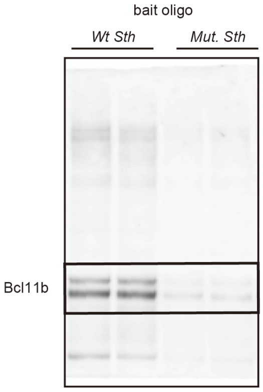

Figure 1b

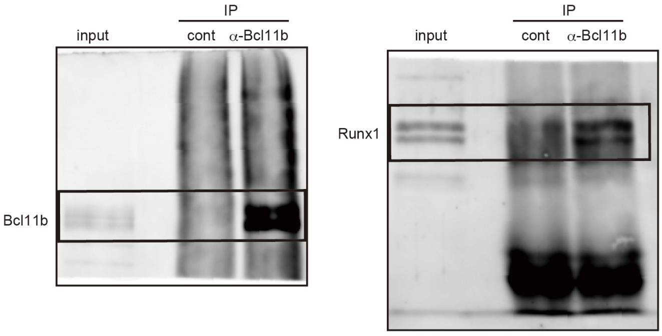

Supplementary Figure 8. Full size images of Immunoblot analyses in Figure 1a and 1b.

Figure 2a

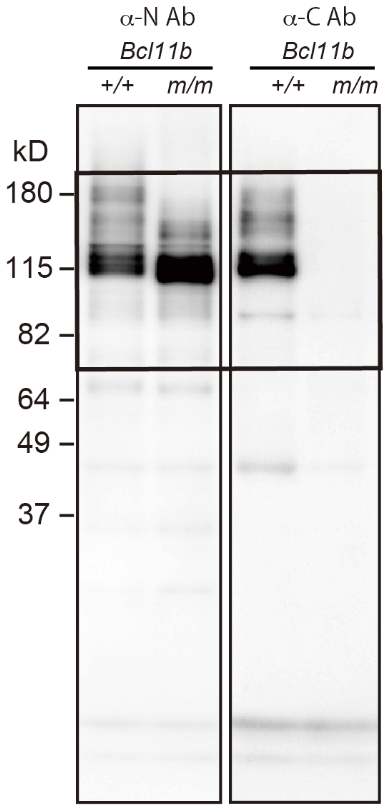

Supplementary Figure 4b

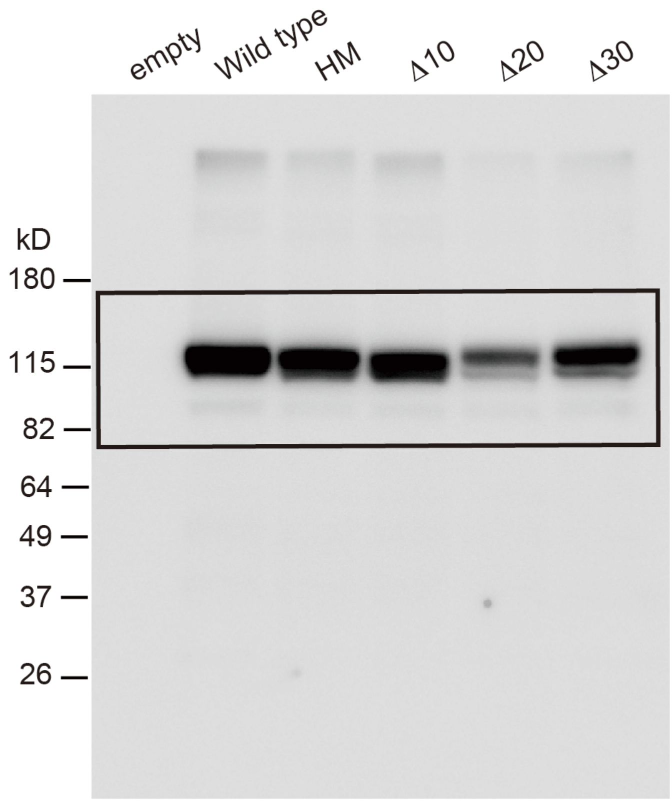

Supplementary Figure 9. Full size images of Immunoblot analyses in Figure 2a and Supplementary Figure 4b.

Supplementary Table 1. Primers for ChIP-qPCR

Target	Forward primer (5'-3')	Reverse primer (5'-3')
Pok-silencer	GGGTAGCACTATTTATAACTGC	AGATCCCAGCGGCGATTAGC
Thpok-PE	CTCCGCCATCTTTATCTTGTTCC	GGTTAAGCTCCCACCAAGTCC
Thpok-intron 1	TGCTGGCCCAGTAAGTTCTG	ATGGCATTGGTGTTGGTACC
URE Sfpi-1	ACTGACCCCTGACACCAAAG	GCCCATCGTGACCTAGAAGA
Foxp3 CNS2	ATCTGGCCAAGTTCAGGTTGTGAC	GGGCGTTCCTGTTTGACTGTTTCT
Foxp3 CNS3	CAGGAAGTGGTTTATGGGTC	ATGAGGATTGGGAGGGGTG
Runx3 -39E	GAAGCTTTTCTCCTGCTCCTC	TATTCACACCCACAGGGAAG
Runx3 -21E	ACAGAGGAAGCAGCATTTGG	TAGCTGTGGGGAACACAAAC
Neg. cont. region	Mouse Negative control primer set 1 (ACTIVE MOTIF: 71011)	

Supplementary Table 2. Primers and probes for qRT-PCR

Target	Forward primer (5'-3')	Reverse primer (5'-3')	Probe
P1-Thpok	AACGAGCAGCGAGCCACTGAC	CTGCTGTGGTCTGGGAATG	FAM-CTGTGCCTCTGCAGCTCCAGCGA-TAMRA
P2-Thpok	TTGCCGGCAAGGCCCCTCAGCGTTC	GAAGTAGTGGCTACAGGCAGCC	FAM-ACCACAGCAGCGAGCTCCTGAGC-TAMRA
P1-Runx3	GTCAGCGTGCGACATGGCTTCCAACAG	AGCACGTCCACCATCGAGCGCACTTCGG	FAM-TGAAGCGGCGGCTGGTGCTC-TAMRA
Socs1	GTGGTTGTGGAGGGTGAGAT	CCTGAGAGGTGGGATGAGG	UPL #20
Socs3	ATTTCGCTTCGGGACTAGC	AACTTGCTGTGGGTGACCAT	UPL #83
Hprt	TCCTCCTCAGACCGCTTTT	CCTGGTTCATCATCGCTAATC	UPL #95
